# Complete Genome Sequence of *Nitrosomonas ureae* Strain Nm10, an Oligotrophic Group 6a Nitrosomonad

**DOI:** 10.1128/genomeA.00094-16

**Published:** 2016-03-10

**Authors:** Jessica A. Kozlowski, K. Dimitri Kits, Lisa Y. Stein

**Affiliations:** Department of Biological Sciences, University of Alberta, Edmonton, Alberta, Canada

## Abstract

The complete genome of *Nitrosomonas ureae* strain Nm10, a mesophilic betaproteobacterial ammonia oxidizer isolated from Mediterranean soils in Sardinia, Italy, is reported here. This genome represents a cluster 6a nitrosomonad.

## GENOME ANNOUNCEMENT

The isolation and genome sequencing of ammonia-oxidizing bacteria (AOB) remain vital to our understanding of the potential roles these organisms play in the global nitrogen cycle. To complement physiological studies on AOB, complete-genome sequences provide insight into how inventory relates to metabolic capacity and environmental niche. The AOB *Nitrosomonas ureae* Nm10 was first isolated from soils in Sardinia, Italy (1), and is an oligotrophic aerobic betaproteobacterium belonging to *Nitrosomonas* cluster 6a (2).

The genome of *N. ureae* was sequenced at the University of Washington, WA, using the PacBio RSII platform; 300,584 raw reads resulted in 166,852 quality-filtered trimmed reads yielding 1,340 Mb, with a mean genome-wide coverage of 311×. The filtered reads were assembled at the University of Alberta, Alberta, Canada, using HGAP version 2.3 (3), and resulted in a 1-contig scaffold. Annotation was performed using the NCBI Prokaryotic Genome Annotation Pipeline (4). The genome is 3.3 Mbp, with a mean G+C content of 44.5% and 2,897 predicted protein-coding genes. The genome includes 40 tRNA genes and a single copy of the 16S-23S-5S rRNA operon. Gene prediction analysis and comparative genomics were performed with IMG (5). The closest neighbor of *N. ureae* is *Nitrosomonas* sp. strain AL212 (6), with an average nucleotide identity (ANI) (7) of 93.18%.

*N. ureae* oxidizes ammonia to nitrite as a sole source of energy and reductant. The genome contains 3 operons for ammonia monooxygenase (*amoCAB*), two of which are followed by the *orf4* and *orf5* genes that are often found in β-AOB (8). Two orphan *amoC* genes were also identified, along with a single copy of the AOB-specific red-copper protein nitrosocyanin (9). It is important to note that this is the first report of an AOB containing four complete operons for hydroxylamine dehydrogenase (*haoAB-cycAB*), as betaproteobacterial AOB usually contain 2 or 3 copies, and one copy often lacks the *cycB* gene (8).

*N. ureae* can utilize urea as an alternate nitrogen source (1) and contains both urea carboxylase (EC 6.3.4.6) and a putative allophanate hydrolase (EC 3.5.1.54) genes (10), as well as genes for a complete urease found in some *Nitrosospira* genomes (11). Carbon fixation genes, including two copies of form I RubisCO-encoding genes, were identified with similarity to those of *Nitrosomonas* sp. strain Is79 (12).

Terrestrial AOB can contribute to nitrogen-oxide release, including the production of nitric and nitrous oxide through nitrifier denitrification (13, 14). The genes in *N. ureae* that are implicated in this process include a copper-containing nitrite reductase (*nirK*), NO-responsive regulator *NnrS*, cytochrome P460 (*cytL*), and cytochrome *c*′ beta (*cytS*). Interestingly, no homologues for nitric oxide reductases were found in the genome, a featured shared by the closely related 6a AOB *Nitrosomonas* sp. Is79 (12).

The *N. ureae* genes for iron acquisition and storage include one copy of the ferric uptake regulation protein (FUR) (15), a *Streptococcus*-like ferric iron ABC transporter (16), two copies of TonB-associated ferric siderophore transporters (17), and two copies of bacterioferritin genes. Two copies of cyanophycin synthetase genes, utilized for nitrogen storage (18), were also identified.

### Nucleotide sequence accession numbers.

The genome sequence has been deposited in GenBank under the accession no. CP013341. The version described in this paper is the first version, CP013341.1.
